# Twin transformation as a strategic approach in sport management: the synergy of digitalization and sustainability in sports

**DOI:** 10.3389/fspor.2024.1403793

**Published:** 2024-07-25

**Authors:** Ekaterina Glebova, Dag Øivind Madsen

**Affiliations:** ^1^ Université Paris-Saclay, CIAMS, Orsay, France; ^2^ Faculty of Business, Higher College of Technology, Dubai, United Arab Emirates; ^3^ Department of Business, Marketing and Law, USN School of Business, University of South-Eastern Norway, Hønefoss, Norway

**Keywords:** twin transition, twin transformation, sustainability, resistance, digitalization, sports, sports management, strategic approach

## Abstract

The integration of digitalization and sustainability principles, encapsulated within the Twin Transformation (TT) approach, has emerged as a transformative paradigm within sport management. However, there is a critical gap in understanding how these two transformative forces can be synergistically harnessed within the field of sport management. This paper explores the conceptual underpinnings of TT and its implications for enhancing organizational performance and addressing contemporary challenges in the sports industry. Drawing upon interdisciplinary perspectives, the paper examines how TT fosters innovation, enhances fan engagement, and promotes environmental responsibility within sport management. Through a synthesis of theoretical insights and practical examples, the paper highlights the potential of TT to drive positive change across various facets of sport management, from operational practices to stakeholder engagement. Furthermore, the paper underscores the importance of ongoing research and scholarly inquiry in advancing our understanding of TT and its implications for theory and practice in sport management. Overall, this paper provides a comprehensive overview of TT in sport management, offering valuable insights for researchers, practitioners, and stakeholders seeking to navigate the dynamic landscape of the sports industry.

## 1 Introduction

Despite extensive research on digitalization and sustainability, there remains a critical gap in understanding how these two transformative forces can be synergistically harnessed within particular fields. Twin Transformation (TT), also known as “twin transition”, embodies a strategic approach that underscores the interconnectedness of digitalization and sustainability in driving organizational change and societal progress (1, 2). Rooted in the recognition that technology and sustainability goals are not mutually exclusive but rather complementary, TT advocates for an integrated approach that leverages digital innovations to advance sustainability objectives and vice versa. This holistic perspective emphasizes the transformative potential of aligning digital and sustainable initiatives to unlock synergies, enhance operational efficiency, and mitigate environmental impact across diverse industries (3). Thus, the concept of TT has emerged as a strategic paradigm with profound implications across various sectors, including sport management (4). Initially conceptualized as a framework that recognizes the potent synergy between technology and sustainability, TT has transcended disciplinary boundaries to become a guiding principle for organizational evolution (5). This article provides an overview of TT in a general context before delving into its application as a perspective and strategic approach specifically tailored for sport management (4).

The problem addressed in this research is the lack of a cohesive framework that integrates digitalization and sustainability within sport management. While previous studies have explored these areas independently, there is a significant research gap in understanding their combined potential to drive organizational change. Within the field of sport management, TT represents more than just a theoretical framework—it serves as a guiding perspective and strategic approach that reshapes how sporting organizations operate in the digital age while embracing sustainability imperatives (6). In this context, TT acknowledges the pivotal role of technology in revolutionizing various facets of the sports industry, from fan engagement and athlete performance (7, 8) to venue management and broadcasting (9). Simultaneously, it emphasizes the responsibility of sports organizations to adopt sustainable practices that minimize ecological footprints and promote social responsibility (10, 11).

By adopting TT as a perspective, sport management professionals gain a nuanced understanding of how digitalization can be harnessed to enhance fan experiences, optimize operational processes, and drive revenue generation in an increasingly competitive landscape (7). Moreover, TT encourages sports organizations to integrate sustainability considerations into their decision-making processes, whether through eco-friendly stadium designs, carbon-neutral event management practices, or community engagement initiatives aimed at promoting environmental awareness.

While reviewing the existing research literature, it became evident that there is a substantial body of work exploring the individual impacts of digitalization and sustainability in various industries. However, there is a noticeable research gap when it comes to the integration of these two transformative forces, particularly within the field of sport management. Previous studies have largely treated digitalization and sustainability as separate domains, resulting in a lack of comprehensive frameworks that address their synergistic potential. This fragmentation has led to missed opportunities for leveraging digital innovations to enhance sustainability efforts and vice versa. Our research addresses these gaps by introducing the concept of TT, which advocates for a holistic approach that intertwines digital and sustainable strategies. By applying TT within sport management, our work demonstrates how digital advancements can be harnessed to achieve sustainability goals, thereby providing a unified framework that enhances operational efficiency, fan engagement, and environmental responsibility. This integrated perspective not only fills the existing research void but also offers practical insights and strategic guidelines for sport organizations seeking to thrive in an increasingly digital and sustainability-conscious world.

Table 1 organizes the article's content into five key areas, providing a clear and systematic overview of the information presented.

**Table 1:** Article contents structure.

Section	Context and problem	Strategic approach	Implementation	Implications
1. Introduction	Critical gap in integrating digitalization and sustainability in specific fields. TT emphasizes the synergy between these two forces for organizational change and societal progress, particularly in sport management.	TT integrates digital innovations to advance sustainability goals. It serves as a strategic paradigm across various sectors, especially sport management, reshaping operations and embracing sustainability.	N/A	TT's holistic perspective aims to enhance operational efficiency and mitigate environmental impact, guiding organizational evolution.
2. TT as a Strategic Approach for Sports Management, Including Research and Innovation Development	Lack of cohesive frameworks in sport management integrating digitalization and sustainability. Previous studies treated them separately, missing synergistic opportunities.	TT provides a roadmap aligning digitalization and sustainability goals, fostering interdisciplinary collaboration for innovative strategies.	TT enhances fan engagement, incorporates sustainability in operations, and leverages technological innovations for competitive advantage.	TT unlocks new revenue streams, mitigates risks, and enhances brand reputation while contributing to environmental and social objectives.
3. TT Application and Guidelines in Sport Management	Importance of integrating digital technologies with sustainable practices in sport management for fan engagement and environmental responsibility.	Steps: Identify stakeholders and objectives, conduct needs assessments, develop cohesive strategies, foster interdisciplinary collaboration, implement digital and sustainable initiatives.	Implementing digital fan engagement initiatives and integrating sustainability into stadium design.	TT approach in sport management can enhance fan experience and promote environmental responsibility.
4. Conclusion	The necessity of TT for contemporary challenges in sport management by intertwining digitalization and sustainability.	TT offers a transformative pathway for sport management, enhancing organizational efficiency and contributing to societal goals.	Calls for further research and practical implementation to refine TT in sport management.	TT represents a comprehensive framework for digitalization and sustainability, providing scientific, policy, and managerial implications.
Scientific Implications	Lack of integrative models in literature combining digitalization and sustainability.	TT provides a novel theoretical model integrating digital and sustainable strategies.	Promotes interdisciplinary research bridging digital innovation and sustainable practices.	Sets a precedent for future studies exploring similar integrative models in other industries.
Policy Implications	Need for regulatory frameworks supporting digitalization and sustainability integration in sports.	Insights for policymakers to develop incentives and regulations for TT adoption.	Collaborative efforts between governmental bodies, sports organizations, and technology providers.	Creates a conducive environment for TT adoption in the sports industry.
Managerial Implications	Fragmented past research focusing on digitalization and sustainability separately.	TT offers a strategic roadmap for operational efficiency and sustainability.	Actionable insights for integrating digital tools with sustainability goals.	Enhances competitive advantage through improved fan engagement and optimized processes.
Limitations of This Study	Sector-specific scope, theoretical focus, and dynamic nature of digital and sustainable innovations.	Continuous adaptation of the TT framework needed.	Limited empirical data to validate practical outcomes.	Strength in integrative approach combining digitalization and sustainability, offering robust theoretical foundation and practical guidelines.
Limitations of Past Research Studies	Focused on digitalization and sustainability as separate entities, lacking comprehensive strategies.	N/A	N/A	It fills these gaps by providing a targeted and actionable TT framework for sports organizations.

## 2 TT as a strategic approach for sports management, including research and innovation development

As a strategic approach, TT provides a roadmap for sports organizations to navigate the complexities of technological innovation and sustainability integration effectively (12). By aligning digitalization and sustainability goals, sport management professionals can unlock new revenue streams, mitigate risks, and enhance brand reputation while contributing to broader environmental and social objectives. Through strategic partnerships, data-driven insights, and stakeholder engagement, sports organizations can leverage TT to stay ahead of the curve and remain resilient in an ever-evolving industry landscape (13).

The application of the TT approach to sport management research and innovation development in sports presents a promising avenue for scholarly exploration and practical implementation (14). At its core, TT advocates for an interdisciplinary approach that synthesizes insights from diverse fields including sport management, technology, sustainability studies, and innovation (7, 15). This integrative perspective offers researchers a comprehensive understanding of the intricate interplay between technology, sustainability, and sports, fostering the emergence of innovative strategies and solutions (16).

One area where the TT approach holds significant potential is in enhancing fan engagement within the sports industry (17, 18). Leveraging digital technologies, such as social media platforms, streaming services, and virtual reality experiences, sports organizations can create immersive fan experiences that transcend geographical boundaries (7). By tapping into these digital platforms, sports entities can amplify audience reach, foster deeper connections with fans, and cultivate a more engaged and loyal fan base (18).

Moreover, the TT approach underscores the importance of integrating sustainability principles into sport management practices (19). In response to growing societal demands for environmental responsibility, sports organizations are increasingly embracing sustainable management practices (20). These initiatives range from implementing green stadium designs and utilizing renewable energy sources to implementing waste reduction strategies (21). By adopting such sustainable practices, sports organizations not only reduce their environmental footprint but also contribute to broader sustainability goals (22).

Technological innovation represents another key aspect of the TT approach in sport management research and innovation development (23, 24). With the rapid advancements in technology, sports organizations have access to a plethora of innovative tools and solutions. From performance tracking devices for athletes to data analytics platforms for coaches and managers, technology offers unprecedented opportunities to enhance performance, optimize training methodologies, and prevent injuries in sports (25). By embracing digitalization and leveraging emerging technologies, sports organizations can drive innovation and maintain a competitive edge in the dynamic sports industry landscape (26).

Ultimately, the adoption of the TT approach in sport management research and innovation development offers a holistic framework for addressing contemporary challenges and driving positive change in the sports industry (27). By integrating digitalization, sustainability, and innovation, sports organizations can unlock new opportunities, improve performance, and create a more sustainable and engaging sporting experience for all stakeholders involved (28, 29).

## 3 TT application and guidelines in sport management

In contemporary sport management, the integration of digital technologies with sustainable practices has become a pivotal strategy for both: enhancing fan engagement and ensuring environmental responsibility (30). By adopting the TT approach, sport management professionals can navigate this intersection effectively, leveraging technology to engage fans while incorporating sustainable principles into stadium design and operations (31).

The first step in applying the TT approach is to identify stakeholders and objectives (32). This includes engaging with various parties such as sport management teams, stadium architects, technology providers, fans, local communities, and environmental organizations (33). The overarching objective is to enhance fan engagement through digital technologies while ensuring sustainability in stadium design and operations (7, 24).

Conducting a comprehensive needs assessment and research is essential to inform the development of the strategy. This involves surveying fans to understand their preferences and expectations regarding digital fan experiences, as well as researching sustainable stadium design principles and best practices in the sports industry (20). Additionally, analyzing technological solutions for enhancing fan engagement, such as mobile apps, augmented reality, and social media integration, provides valuable insights for strategy development (34).

Developing a cohesive strategy is crucial for aligning digital fan engagement with sustainable stadium design. This entails creating a strategic plan that outlines how digital fan engagement initiatives will be integrated with sustainable stadium design principles. Clear goals and timelines for implementation should be established, taking into account budgetary constraints and resource availability. Key performance indicators (KPIs) should also be defined to measure the success of the initiative, including fan satisfaction ratings, energy efficiency metrics, and waste reduction targets.

Collaboration across disciplines is essential for the successful implementation of the strategy. This involves fostering collaboration between sport management professionals, stadium architects, technology experts, and sustainability specialists. Interdisciplinary brainstorming sessions can generate innovative ideas and solutions that leverage the strengths of each discipline, ensuring a holistic approach to digital fan engagement and sustainability in stadium design.

Implementation of digital fan engagement initiatives involves developing and deploying technological solutions to enhance the fan experience (9). This may include creating a mobile app that provides fans with interactive experiences, installing digital signage and displays throughout the stadium, and leveraging social media platforms for engagement before, during, and after events (7).

Integrating sustainability into stadium design requires thoughtful planning and execution (35). This involves designing the stadium with sustainability in mind, incorporating features such as renewable energy sources, water conservation systems, and eco-friendly materials. Additionally, waste management strategies should be implemented to minimize environmental impact, including recycling bins, composting facilities, and food donation programs (36). Monitoring and evaluating performance is essential for optimizing outcomes and achieving long-term success. This involves regularly monitoring KPIs to assess the impact of digital fan engagement initiatives and sustainability efforts (10). Soliciting feedback from fans, staff, and other stakeholders helps identify areas for improvement and innovation, allowing for adjustments to strategies and tactics based on performance data and stakeholder input (37).

By following this guideline, sport management professionals can effectively apply the TT approach to integrate digital fan engagement with sustainable stadium design, ultimately enhancing the overall fan experience while promoting environmental responsibility, accessibility and inclusion within the industry (34, 38).

The implementation of the TT approach in sport management is exemplified by the case of U.S. Bank Stadium, home to the Minnesota Vikings football team (39). This stadium provides a tangible illustration of how digital fan engagement can be seamlessly integrated with sustainable stadium design principles to enhance the overall fan experience while promoting environmental responsibility.

At the outset, various stakeholders including the Minnesota Vikings organization, stadium architects, technology providers, fans, local community representatives, and environmental organizations were identified. The primary objectives were twofold: to enhance fan engagement through digital technologies and to ensure sustainability in stadium design and operations.

To inform the strategy development process, the Minnesota Vikings conducted extensive needs assessments and research endeavors. Surveys and focus groups were conducted to gain insights into fan preferences regarding digital fan experiences. Concurrently, research was undertaken to explore sustainable stadium design principles, encompassing energy-efficient building materials, waste management strategies, and eco-friendly operational practices.

Subsequently, a comprehensive strategy was devised to integrate digital fan engagement initiatives with sustainable stadium design features. This involved setting clear goals and objectives, delineating strategies for fan engagement via mobile applications, digital signage, and social media integration, alongside outlining plans for sustainability enhancements such as energy-efficient lighting, water conservation measures, and waste reduction initiatives.

Interdisciplinary collaboration and stakeholders' cooperation (16) seem to play a pivotal role in the implementation process. The Minnesota Vikings collaborated closely with stadium architects, technology experts, and sustainability specialists to develop innovative solutions that harmonized digital fan engagement with sustainable design principles, ensuring a holistic and cohesive approach to implementation.

Implementation of digital fan engagement initiatives involved the development of a mobile application offering interactive experiences, installation of digital signage throughout the stadium, and leveraging social media platforms for fan interaction and communication (35). Concurrently, sustainable design features were integrated into the stadium's infrastructure, encompassing energy-efficient lighting systems, rainwater harvesting mechanisms, and waste management facilities (36). Throughout the implementation process, rigorous monitoring and evaluation mechanisms were employed to assess performance and measure impact, KPIs and feedback are involved in techniques.

The case of U.S. Bank Stadium is a simplified illustration of how the TT approach can be effectively applied in sport management. By integrating digital fan engagement initiatives with sustainable stadium design principles, the Minnesota Vikings organization not only enhanced the fan experience but also demonstrated a commitment to environmental responsibility and sustainability within the sporting domain.

## 4 Conclusion

The TT approach emerges as a pivotal paradigm within sport management, offering a comprehensive framework for the integration of digitalization and sustainability principles. Through this convergence, TT holds significant promise for reshaping various dimensions of sport management, including operational practices, stakeholder engagement, vision and philosophy, and environmental stewardship.

The discourse presented underscores the critical importance of TT in navigating contemporary challenges within the sporting domain. By embracing digitalization and sustainability as intertwined elements, sport management professionals stand to unlock synergistic advantages and drive transformative change. This strategic alignment not only enhances organizational efficiency and competitiveness but also contributes to broader societal and environmental goals.

Moving forward, it is imperative for researchers and practitioners alike to further develop and refine the concept of TT within the realm of sport management. Through rigorous academic inquiry and practical implementation, deeper insights can be gained into the mechanisms and impacts of TT on organizational performance and societal outcomes. Such endeavors not only advance scholarly understanding but also inform evidence-based practices that drive positive change within the sports industry.

To this end, the TT approach represents a transformative pathway for sport management, characterized by its emphasis on digitalization, sustainability, and holistic integration. By embracing TT as a guiding principle, researchers and practitioners can navigate the evolving landscape of sport management with foresight and purpose, ultimately contributing to the advancement of both theory and practice in the field.

## 5 Scientific implications

This perspective significantly contributes to the scientific understanding of the interconnectedness between digitalization and sustainability within the context of sport management. By introducing and applying the TT framework, this research provides a novel theoretical model that integrates digital and sustainable strategies, offering a comprehensive approach that had been previously overlooked. This interdisciplinary approach not only bridges the gap between digital innovation and sustainable practices but also sets a precedent for future studies to explore similar integrative models in other industries.

## 6 Policy implications

From a policy perspective, the paper highlights the necessity for regulatory frameworks and policy initiatives that promote and support the integration of digitalization and sustainability in the sports industry. Policymakers can use the insights gained from this study to develop incentives for sports organizations to adopt TT principles, such as subsidies for green technology investments or regulations mandating sustainable practices. Additionally, this perspective underscores the importance of collaborative efforts between governmental bodies, sports organizations, and technology providers to create a conducive environment for TT adoption.

## 7 Managerial implications

For managers and practitioners within the sports industry, the practical application of TT offers a strategic roadmap for enhancing both operational efficiency and sustainability. By implementing TT, sports organizations can achieve a competitive advantage through improved fan engagement, optimized processes, and sustainable practices. This research provides actionable insights for managers on integrating digital tools to support sustainability goals, such as using data analytics for energy management in stadiums or leveraging social media platforms to promote environmental awareness among fans.

## 8 Limitations of this study

Despite its contributions, this study has several limitations. First, the scope of the research was confined to the sports industry, which may limit the generalizability of the findings to other sectors. Second, the implementation of TT principles was examined primarily through a theoretical lens, with limited empirical data to validate the practical outcomes. Finally, the dynamic nature of digital and sustainable innovations means that the framework proposed may need continuous adaptation to remain relevant in the face of emerging technologies and evolving sustainability standards. On the other hand, one of the key strengths of this research is its integrative approach, combining digitalization and sustainability into a cohesive framework that addresses a significant gap in the literature. The comprehensive review and synthesis of existing studies provide a robust theoretical foundation for TT. Additionally, the strategic guidelines and practical implications outlined in the study offer valuable tools for practitioners and policymakers, enhancing the practical relevance and applicability of the research findings.

## 9 Limitations of past research studies

Previous research studies have primarily focused on digitalization and sustainability as separate entities, failing to explore their potential synergies. This fragmented approach has resulted in a lack of comprehensive strategies that leverage the interdependent benefits of digital and sustainable innovations. Furthermore, past studies often lacked a sector-specific focus, making it challenging to apply their findings to specific industries such as sport management. By addressing these limitations, our research not only fills the existing gaps but also provides a targeted and actionable framework that can be readily adopted by sports organizations.

## Data Availability

The original contributions presented in the study are included in the article/Supplementary Material, further inquiries can be directed to the corresponding authors.
